# Snellenius manilae bracovirus suppresses the host immune system by regulating extracellular adenosine levels in *Spodoptera litura*

**DOI:** 10.1038/s41598-020-58375-y

**Published:** 2020-02-07

**Authors:** Yuan Chang, Cheng-Kang Tang, Yu-Hsien Lin, Chih-Hsuan Tsai, Yun-Heng Lu, Yueh-Lung Wu

**Affiliations:** Department of Entomology, National Taiwan University, Taipei, 106 Taiwan

**Keywords:** Signal transduction, Pathogens

## Abstract

Sufficient energy supply to the host immune system is important for resisting pathogens. Therefore, during pathogen infection, the host metabolism is reassigned from storage, growth, and development to the immune system. Previous studies in *Drosophila melanogaster* have demonstrated that systemic metabolic switching upon an immune challenge is activated by extracellular adenosine signaling, modulating carbohydrate mobilization and redistributing energy to the hemocytes. In the present study, we discovered that symbiotic virus (SmBV) of the parasitoid wasp *Snellenius manilae* is able to down-regulate the extracellular adenosine of its host, *Spodoptera litura*, to inhibit metabolism switching. The decreased carbohydrate mobilization, glycogenolysis, and ATP synthesis upon infection results in the host being unable to supply energy to its immune system, thus benefitting the development of wasp larvae. When we added adenosine to the infected *S. litura* larvae, we observed enhanced host immune responses that decreased the pupation rate of *S. manilae*. Previous studies showed that after pathogen infection, the host activates its adenosine pathway to trigger immune responses. However, our results suggest a different model: we found that in *S. manilae*, SmBV modulates the host adenosine pathway such that wasp eggs and larvae can evade the host immune response.

## Introduction

The immune response is an energy-demanding process. When vertebrates are infected by pathogens, host immune responses are activated, which are intimately associated with metabolic switching including the redistribution of energy supply and increased glycolysis and glucose consumption in the immune system^1,2^. This metabolic reprogramming has also been observed in *Drosophila*, whereby delayed development and increased carbohydrate mobilization (hyperglycemia) was found after infection with parasitoid wasps or bacteria^3,4^. These phenomena are due to the fact that, during infection, the energy normally used for larval development and stored in the fat body is redistributed in order to supply the immune system for hemocyte differentiation and cellular immune responses such as phagocytosis and encapsulation. During an immune challenge, immune cells must respond rapidly to pathogens and adjust their own metabolism; a similar phenomenon in mammalian systems is termed the Warburg effect^5^. In *Drosophila*, this metabolism switching process has been proven to be regulated by extracellular adenosine signaling^6^.

Adenosine is a known signaling molecule regulating several cellular processes. Under stress conditions, increased mitochondrial activity increases ATP content, which is subsequently converted into adenosine and transported to the extracellular environment through nucleoside transporters^7^. Extracellular adenosine binds to adenosine receptors (AdoR) and modulates downstream PKA/cAMP signaling; in *Drosophila melanogaster* this has been reported to regulate neurological functions, cell growth, hematopoiesis, and metabolic switching^4,8–11^. In addition, dysregulation of extracellular adenosine signaling interrupts the carbohydrate metabolism, hemocyte proliferation and ecdysone synthesis; the metabolic enzyme, adenosine deaminase-related growth factors (ADGFs; also known as ADA2 in mammals) plays an important role in maintaining extracellular adenosine homeostasis upon wasp or bacterial infection in *Drosophila*^3,12–14^.

*Snellenius manilae* belongs to the order Hymenoptera and family Braconidae and is an endoparasitic wasp with high host specificity^15,16^. The host of *S*. *manilae* is mainly larvae of the family Noctuidae. *S. manilae* contains symbiotic polydanviruses (PDVs); these inhibit the host immune response and help promote wasp development in the host^17^. PDVs are circular and double-stranded DNA viruses belonging to the Polydnaviridae family^18–20^. According to its host, PDVs are divided into two genera: bracoviruses (BVs) and ichnoviruses (IVs)^21,22^. Their genomes are 190–500 kb in length and divided into different segments that are packaged by the capsid to form viral particles with different gene segments. The main function of PDVs in wasps is to protect their progeny from the host immune system. During wasp oviposition, PDV particles enter the host along with eggs and the host expresses the viral genes^21^. These viral genes have three functions: (i) modifying host growth and metabolism to provide energy to the wasp larvae^23^, (ii) inhibiting host metamorphosis, and (iii) host immune responses^24,25^.

Adenosine can act as a signal for metabolic switching such that the energy required for growth and development is transferred to the immune system to activate immune responses. However, when *S. litura* is parasitized by *S*. *manilae*, the spread of PDV (Snellenius manilae bracoviruses, SmBVs) attenuates the host immune response. Previous studies have confirmed that the virus directly inhibits immune gene expression^26^. The results of our study indicate that SmBVs inhibit transcriptions of the host adenosine receptor, transporters, and metabolic enzymes as well as reduce the adenosine level, resulting in an effect on the host carbohydrate metabolism, which is required for immune responses. These results prove that SmBVs regulate adenosine and energy transport to affect host immune responses. Previous studies showed increased adenosine and circulative glucose levels after pathogen entry activate immune responses, whereas our study shows different results: after insects are infected by SmBVs, adenosine, glucose, glycogenolysis and ATP synthesis are attenuated, resulting in a loss of immune responses.

## Results

### Immune suppression by parasitoid symbiont virus, SmBV, via extracellular adenosine signaling

**Figure 1:**
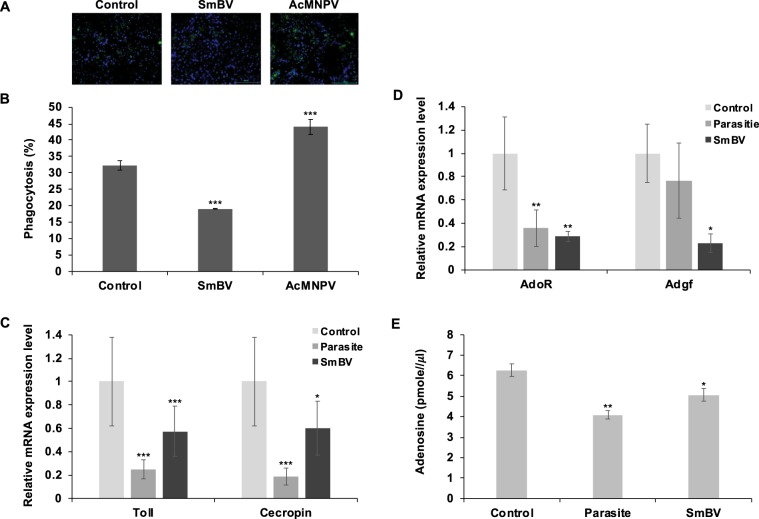
Suppression of immune responses and disruption of adenosine signaling after SmBV or wasp infection in *S. litura*. (**A**) Phagocytic capacity of hemocytes from fourth-instar larvae was measured 36 h after infection with SmBV (1 × 10^5^ copies/larva) or AcMNPV (1 × 10^5^ copies/larva). Green fluorescence: *E. coli*, blue fluorescence: nucleus. (**B**) Results of fluorescence quantitation . (**C**) qPCR detection of *Toll-7* and *Cecropin* expression levels. (**D**) RT-qPCR analyses of *AdoR* and *Adgf* expressions in the second-instar *S. litura* larvae infected by wasp or SmBV. Expression levels of *AdoR* and *Adgf* were analyzed 36 hours post-infection. (**E**) Measurement of adenosine levels in the hemolymph of *S. litura* after wasps or SmBV infections. All values are shown as the mean ± SD of three replicates for qPCR and four replicates for adenosine measurement (*p-value < 0.05, **p-value < 0.01; ***p-value < 0.005).

Previous studies have shown that PDVs inhibit the cellular^27^ and humoral immunity^28–30^ of the host. Therefore, we tested the effects of SmBV on cellular immunity in *S. litura* by phagocytosis. Results showed that the hemocytes collected from SmBV-infected larvae exhibited decreased phagocytic activity compared with the control, indicating attenuated cellular immunity after SmBV infection (Fig. 1A). *S. litura* is a natural host for AcMNPV, and AcMNPV was used as a control. Results showed that phagocytosis increased in AcMNPV-infected larvae, indicating that AcMNPV infection induces cellular immune response. Phagocytosis quantitation results showed that only 19% of the hemocytes from SmBV-infected larvae phagocytized FITC-labeled *E. coli* in comparison to 32% of non-infected larvae and 40% of AcMNPV-infected larvae (Fig. 1B). Previous studies reported that viruses predominantly activate humoral immunity via the Toll-7 pathway^31^. Therefore, we quantified the gene expression levels of the Toll-7 receptor and a downstream antimicrobial protein (Cecropin) after infections. Results showed that gene expression levels of *Toll* and *Cecropin* decreased in *S. manilae*-parasitized or SmBV-infected *S*. *litura* (Fig. 1C), suggesting that immune responses of *S*. *litura* are inhibited by SmBV.

Previous studies have demonstrated that extracellular adenosine signaling is up-regulated upon pathogenic infections. Hence, to confirm whether extracellular adenosine signaling is affected in *S. litura* after SmBV infection, we first measured *AdoR* and *Adgf* expressions, which indicate the status of GPCR signaling and adenosine metabolism, respectively. We found that the gene expression levels of *AdoR* and *Adgf* were reduced in *S. manilae*-parasitized or SmBV-infected hosts (Fig. 1D). As an extracellular adenosine deaminase, expression of *Adgf* is tightly modulated following the titer of extracellular adenosine^13,32^. Hence, decreased *Adgf* expression indicates that extracellular adenosine might be decreased after SmBV infection. To confirm this, we quantified extracellular adenosine levels in the hemolymph of infected larvae. The adenosine level in the hemolymph of the controls was found to be 6.24 pmol/μl; these levels were significantly lower in SmBV-infected and *S. manilae-*parasitized larvae, 5.47 pmol/μl and 4.7 pmol/μl, respectively (Fig. 1E). These results indicate that the adenosine signaling of *S. litura* is influenced by SmBV infection.

### Down-regulation of systematic metabolism switching by wasp and SmBV infections

**Figure 2:**
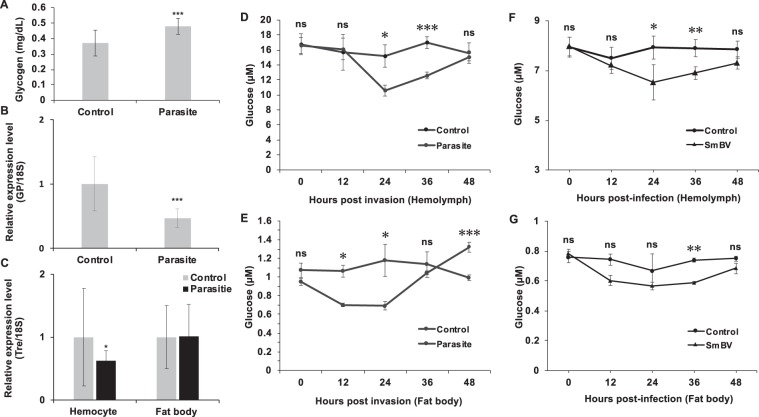
Carbohydrate level in the hemolymph of *S. litura* is affected after parasitization by *S. manila*. (**A**) Changes in glycogen level in fat body of the second-instar larvae 36h post-infections (n = 8, ***p-value < 0.005). qPCR analyses of glycogen phosphorylase (*GP*) (**B**) and trehalase (*Tre*) (**C**) expressions in the second-instar *S. litura* larvae. Expression levels of *GP* and *Tre* were measured 36 hours post-infection. Measurement of the glucose level in the (**D**) hemolymph and (**E**) fat body of second-instar larvae 0, 12, 24, 36, and 48 h after wasp parasitization. Measurement of the glucose level in the (**F**) hemolymph and (**G**) fat body of fourth-instar larvae 0, 12, 24, 36, and 48 h after SmBV infection (1 × 10^5^ copies/larva). All values are shown as the mean ± SD of three replicates for qPCR. (*p-value < 0.05, ***p-value < 0.005, ns: non-significant).

Since adenosine signaling is a known pathway for regulating energy switching from development and storage toward immune response upon an immune challenge^4^, and based on the results in the previous section, we hypothesized that the reduction in adenosine level by SmBV infection might alter the host metabolism, resulting in inhibition of the immune responses of *S. litura*. Quantification of the glycogen levels in the fat body showed that it increased to 0.48 mg/dl after parasitization by *S*. *manilae* (Fig. 2A). Decreased glycogen phosphorylase (*glyp*) expression also indicated that glycogenolysis is not activated after wasp parasitization (Fig. 2B). These results suggest that glycogenesis remained in the fat bodies of parasitized hosts. In addition, expression of trehalase (*tre*) in the hemocytes was decreased, while it remained unchanged in the fat body (Fig. 2C). These results suggest that glycolysis in the hemocyte of infected larvae was down-regulated by *S. manilae* parasitization. In addition, the downregulation of carbohydrate mobilization was also shown in wasp- and SmBV-infected larvae. The glucose level in hemolymph decreased 24 h after parasitization, and the lowest level was detected after 24 h (Fig. 2D). The glucose level in fat body decreased 12 h post-parasitization and reached the lowest level at 24 h (Fig. 2E). These results indicate that circulative glucose in hemolymph decreases after parasitization by *S. manilae*, resulting in a reduction in glucose supply to immune cells in the hemolymph for immune responses. To further confirm that SmBV alone was responsible for the reduced glucose supply in the hemolymph, SmBVs were injected into fourth-instar larvae; it was found that the glucose levels decreased significantly in the hemolymph (Fig. 2F) and fat body (Fig. 2G). These results demonstrate that SmBVs play a key role in inhibiting carbohydrate mobilization in the host.

**Figure 3:**
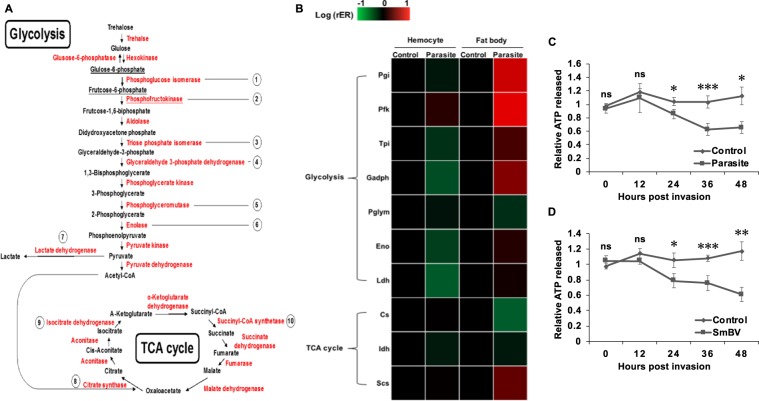
SmBV infection affects gene expression of enzymes involved in glycolysis and TCA cycle in hemocytes. (**A**) Glycolysis and TCA cycle metabolic pathways. Numbers are the genes quantitated in qPCR. (**B**) qPCR was used to quantitate the gene expression levels of metabolic enzymes in hemocytes and fat body. Ct values were obtained and standardized, and Excel was used to plot the heat map. Red represents an increase in gene expression level, while green represents a decrease in gene expression level. The quantitated metabolic enzymes include Pgi, Pfk, Tpi, Gapdh, Pglym, Eno, Ldh, Cs, Idh, and Scs. All values are shown as the mean ± SD of three replicates. The ATP level was measured in the hemolymph of second-instar larvae 0, 12, 24, 36, and 48 h after wasps (**C**) or SmBV infection (**D**) (1 × 10^5^ copies/larva). All values are shown as the mean ± SD of four replicates for ATP measurements, and *P*-values were calculated using Student’s *t*-test (*p-value < 0.05, **p-value < 0.01; ***p-value < 0.005).

Previous studies using *Drosophila* infected by wasps or bacteria demonstrated that adenosine signaling initiates glycogen breakdown in the fat body and increases carbohydrate mobilization in the hemolymph during an immune challenge^3,4^. Moreover, the expression of glycolytic genes was shown to be decreased in the fat body but increased in hemocytes; this metabolic switching process is critical for supplying sufficient energy to the hemocytes. Notably, and unlike in the *Drosophila* model, our results reveal an increased glycogen level and decreased carbohydrate mobilization in *S. litura* after wasp *S. manilae* or SmBV infection (Fig. 2). To compare our results, we further measured the expression of glycolytic and TCA cycle genes in the hemocyte and fat body of *S. litura* after *S. manilae* parasitization (Fig. 3A). The expression levels of glycolytic and TCA enzymes did not show a significant change or even a decrease after parasitization (Fig. 3B). Conversely, the expression levels of the same set of metabolic genes were increased in the fat body. These results indicate that metabolic switching does not initiate after *S. manilae* parasitization, so the energy distribution is not switched toward immune responses. In addition, we also found that the ATP titer was decreased after *S. manilae* parasitization (Fig. 3C) or SmBV infection (Fig. 3D), indicating that ATP synthesis is suppressed by SmBV infection. Since extracellular adenosine could be converted from extracellular ATP, these results correspond to our observation of decreased extracellular adenosine titer after SmBV infection (Fig. 1E).

### Carbohydrate metabolism regulated by extracellular adenosine signaling is required for immune response

**Figure 4:**
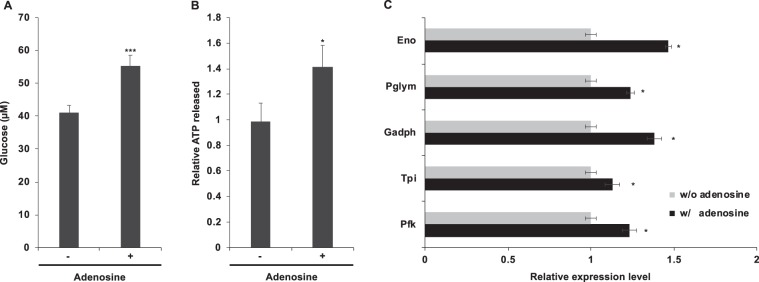
Addition of adenosine increases carbohydrate metabolism. Analysis of glucose (**A**) and ATP (**B**) levels in the hemolymph of fourth-instar larvae after feeding with artificial diet containing 5 mM adenosine. (**C**) qPCR was used to quantify the gene expression levels of metabolic enzymes in hemocytes of fourth-instar larvae after feeding with 5 mM adenosine. At least three repetitions were conducted for each group. All values are shown as the mean ± SD, and *P*-values were calculated using Student’s *t*-test (*p-value < 0.05).

**Figure 5:**
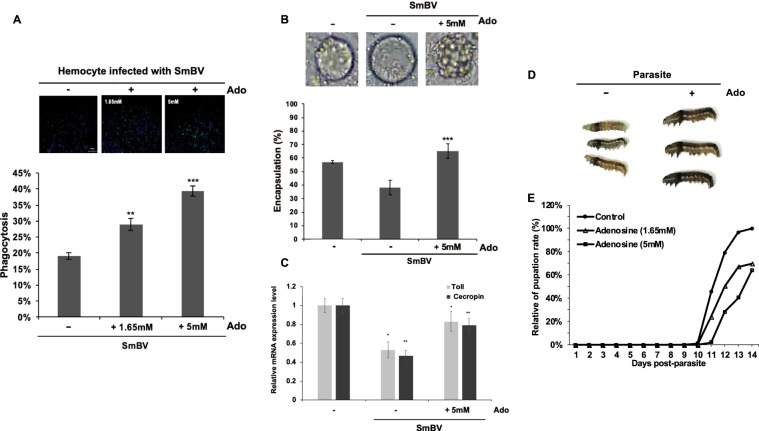
Addition of adenosine increases immune responses in *S. litura*. Phagocytosis (**A**) and encapsulation (**B**) assays of the hemocytes in the fourth-instar larvae which were fed artificial diet containing 1.65 mM or 5 mM adenosine for 1 week and infected with SmBV (1 × 10^5^ copies/larva) for 36 h. Upper panel: images of phagocytosis or encapsulation; lower panel: quantitation results. (**C**) qPCR detection of *Toll-7* and *Cecropin* expression levels in infected larvae feeding wtih 5 mM adenosine. All values are shown as the mean ± SD of three replicates, and *P*-values were calculated using Student’s t-test (*P < 0.0, **P < 0.005, ***p-value < 0.005). (**D**) Newly hatched *S. litura* larvae were fed with artificial diet containing 5 mM adenosine. Wasp infection were conducted 48 h after *S. litura* had molted to become second-instar larvae. Growth status of *S. litura* fed with artificial diet containing or not containing adenosine after wasp parasitization. (**E**) Percentage of wasp pupariation. The pupation rate of the control was set as 100% for comparison with the pupation rate after 1.65 mM or 5 mM adenosine treatment. All experiments were performed with three replicates.

Our results implied that inhibition of the host immune response after SmBV infection might be due to suppression of its carbohydrate metabolism by the down-regulation of adenosine levels. To confirm the effect of adenosine on carbohydrate metabolism, insects were fed with 5-mM adenosine; the glucose level (Fig. 4A) and ATP level (Fig. 4B) in hemolymph were found to be higher than that of the control. In addition, the expression levels of the genes involved in glycolysis and the TCA cycle showed significantly increased in the hemocytes after feeding with adenosine, indicating that adenosine promotes carbohydrate metabolism (Fig. 4C). To confirm whether adenosine affects immune responses, SmBV-infected fourth-instar larvae were fed with adenosine at concentrations of 1.65 mM and 5 mM, and their hemocytes subjected to a phagocytosis assay with FITC-labeled *E. coli*. Results showed that phagocytic activity increased in SmBV-infected larvae given 1.65 mM or 5 mM adenosine compared to control larvae (Fig. 5A). Quantitation results showed that the proportion of phagocytosis increased in larvae given 1.65 mM and 5 mM adenosine by 10% and 20%, respectively, compared with larvae that were infected with virus only. We further tested the encapsulation activity of the hemocytes by using beads after adenosine treatment, and the results showed that the encapsulation activity was significantly increased by 35% in larvae given 5 mM adenosine (Fig. 5B). Moreover, the suppression of *Toll-7* and *Cecropin* expressions by SmBV infection were restored after feeding with 5 mM adenosine (Fig. 5C). These results demonstrate that adenosine induces cellular and humoral immune response in SmBV-infected larvae. The growth inhibition by wasp parasitization was also restored after adenosine treatment, suggesting that artificial feed containing adenosine was able to suppress wasp infection (Fig. 5D). In addition, wasp pupation decreased by 40% compared with the control when 5 mM adenosine was given to *S. litura* (Fig. 5E). These results confirm that decreased adenosine signaling by SmBV or wasp infection causes the immune suppression, and feeding with adenosine can reverse this immune suppression.

### SmBV negatively affects host adenosine pathway

**Figure 6:**
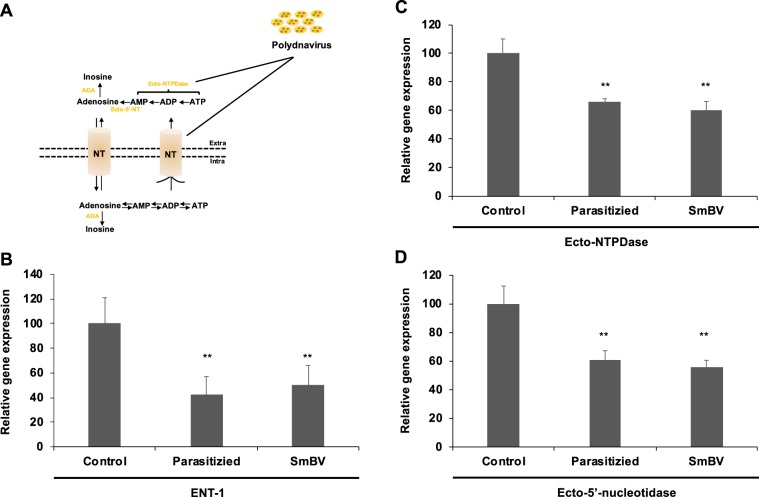
Inhibition of the gene expressions associated with the adenosine metabolism pathway by wasp infection. (**A**) Schematic diagram of adenosine synthesis and metabolic pathways. qPCR was used to quantitate the gene expression levels of *ENT1* (**B**), *Ecto-NTPDase* (**C**), and *Ecto-5*′*-nucleotidase* (**D**) in the second instar larvae after wasp infection. At least three repetitions were performed. Theexpression level of control group was set to 100%. *P*-values were calculated using Student’s t-test (*P < 0.0, **P < 0.005).

To investigate how SmBV decreases extracellular adenosine levels, we quantitated the gene expression levels of the enzymes involved in adenosine metabolism pathway in SmBV-infected larvae, including Ecto-NTPDase and Ecto-5′-nucleotidase, as well as the adenosine transporter, equilibrative nucleoside transporter (ENT) (Fig. 6A). Results showed that the gene expression levels of *Ecto-NTPDase*, *Ecto-5*′*-nucleotidase*, and *ENT1* (Fig. 6B–D) were lower in SmBV-infected larvae compared to the control. This showed that the enzymes for maintaining the adenosine homeostasis were affected after SmBV infection, thus resulting in reducing the extracellular adenosine concentration in infected larvae.

### Effects of adenosine pathway and energy synthesis inhibitors on host immune responses

**Figure 7:**
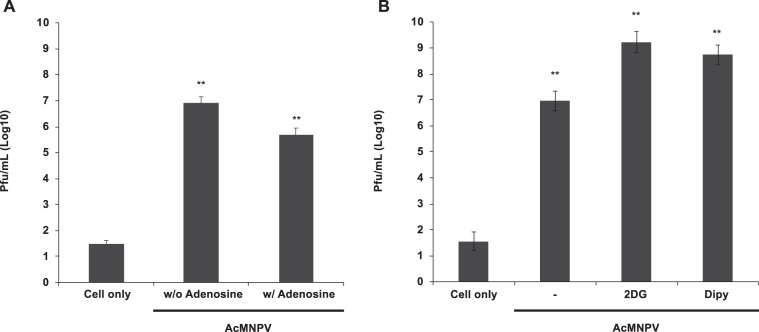
Differences in AcMNPV titers after SL1A cells were treated with adenosine, adenosine transport inhibitor (Dipy) and glycolysis inhibitor (2DG). Virus titers were measured in AcMNPV infected SL1A cells with and without the adding of adenosine (**A**), 2DG, or Dipy. (**B**) The virus titers were estimated at 48 hours post-infection. “Cell only” indicates cells without virus and drug treatments. All values are shown as the mean ± SD of three replicates. *P*-values were calculated using Student’s *t*-test (*P < 0.0, **P < 0.005).

Our results show that the application of additional adenosine increases both cellular and humoral immune responses which are suppressed by SmBV infection. Since adenosine is an important signal for activating the immune response via modulating the host energy metabolism, we further examined whether inhibition of adenosine signaling and energy synthesis compromise immune responses upon infection. As SmBV is not able to replicate in the host, we used AcMNPV-infected *S. litura* cell lines to analyze the effects. Our result showed that the AcMNPV titer significantly decreased in infected-cells treated with adenosine (Fig. 7A). Conversely, the applications of the glycolysis or adenosine transportation inhibitors, 2-deoxy-D-glucose (2DG) and dipyridamole (Dipy) increased the virus titers significantly (Fig. 7B). 2DG is a glucose analog and is considered as a competitive inhibitor of glycolysis. In cells, 2DG competes with glucose for hexokinase. However, 2DG forms 2-DG-6-phosphate, which cannot undergo glycolysis and affects energy production. Dipy is an inhibitor of nucleoside transporters on the cell membrane that can inhibit adenosine transport between cells. Our experiments proved that suppression of adenosine transportation as well as glycolysis of SL1A cells compromise the antivirus responses. Together with our pervious *in vivo* experiments, we conclude that activation of adenosine signaling is essential for metabolism reprogramming which is important for host antivirus responses. The SmBV is capable of suppressing the adenosine signaling in *S. litura* resulted in a failure of energy switching and decreased host antivirus responses.

## Discussion

Our results showed that phagocytosis and *Toll* and *Cecropin* gene expression are suppressed after wasp and SmBV infection (Fig. 1); these results correspond to previous studies proving that PDV infection modulates the host growth and metabolism^22,23,33^ as well as inhibiting cellular and humoral immune responses^27–29,34^. Several studies have demonstrated the importance of adenosine in regulating physiological reactionsupon cell damage and pathogen infection in mammals and *Drosophila*^4,35–38^, and it can act as a signal for metabolic switching to provide energy to the immune system and/or cell repair. The function of adenosine in mammals is to modulate the inflammatory and immune processes; ischaemia, hypoxia, inflammation, and oxidative/nitrosative stress can all stimulate its release from any type of cell^39^. In *Drosophila*, it was found that pathogen infection increased adenosine levels^40^, leading to increased glucose and trehalose levels to provide energy to hemocytes to differentiate into functional immune cells to activate immune responses^4,37^. Our study revealed that after *S. litura* was infected by SmBV, its extracellular adenosine level decreased and the gene expression levels of *AdoR* and *Adgf* decreased significantly (Fig. 1D,E). It has been described that blocking AdoR signaling under non-infectious conditions decreases prohemocyte differentiation in the lymph gland^11^. Taking together, decreasing in adenosine signal might be one of the strategies for SmBV to suppress the host immune response.

In the present study, our resutls showed that glycogen level was higher and expression of *GP* was lower in the fat body of SmBV-infected larvae in comparing to non-infected larvae (Fig. 2A). This indicates that the efficiency of converting glycogen to glucose in the *S. litura* fat body is reduced after SmBV infection^41,42^. In addition, the glucose content in the hemolymph was significantly lower after infection (Fig. 2D). Previous studies on *Galleria mellonella* showed decreased carbohydrate levels in *G. mellonella* pupae 24 h after parasitization with PDV-containing *Pimpla turionellae*^43^. A similar phenomenon was also observed in SmBV-infected *S. litura*. However, after *S. litura* was infected by SmBV, the glucose level in both hemolymph and fat bodies was restored after 48 h (Fig. 2D,E). Restoration of the carbohydrate level in the host after initial suppression upon infection may be essential for the growth and development of wasp larvae^44^.

In the present study, the extracellular adenosine level was found to decreases after SmBV infection of *S. litura*; this suppression leads to reduced immune responses as demonstrated by a phagocytic activity assay of the hemocytes (Fig. 1A). While oral administration of adenosine in infected *S. litura* increased the phagocytic activity (Fig. 4A). Oral administration was chosen in this study because injection could cause injury, which would interfere with the subsequent physiological analyses. To confirm that the orally-administered adenosine reached the hemolymph, the amount of adenosine in hemolymph was measured; it was confirmed to increase significantly (data not shown).

Adenosine synthesis mainly occurs through two pathways. The first pathway occurs when cells are under metabolic stress (reduction in the intracellular ratio of ATP and AMP)^45,46^, where ATP in cells is metabolized to form adenosine which may further be transported to extracellular space via ENTs^47,48^. The second pathway occurs when ATP/ADP are released from damaged cells upon stresses, they will be converted to adenosine through Ecto-NTPDase and Ecto-5′-NT^49^. However, when *S. litura* was infected by SmBV, it was found that not only extracellular ATP levels (Fig. 3C,D) but also expression levels of *Ecto-NTPDase*, *Ecto-5*′*-NT*, and transporter *ENT1* are decreased (Fig. 6B–D), indicating that SmBV affects host adenosine metabolism. At present, it is still unclear how SmBV affects the host adenosine pathway. Previous studies have indicated that PDVs contain many non-coding regions that can produce miRNAs^50,51^, and that these miRNAs can regulate the host physiological responses^52,53^. Preliminary prediction results of SmBV miRNAs showed that seven miRNAs produced by SmBVs may be capable of inhibiting enzymes involved in the adenosine pathway (Fig. S1). It is therefore hypothesized that SmBVs may produce miRNAs to modulate adenosine metabolic genes of the host, which may contribute to a decreased adenosine level in SmBV-infected *S. litura*. Further experiments will be performed to test this hypothesis.

**Figure 8:**
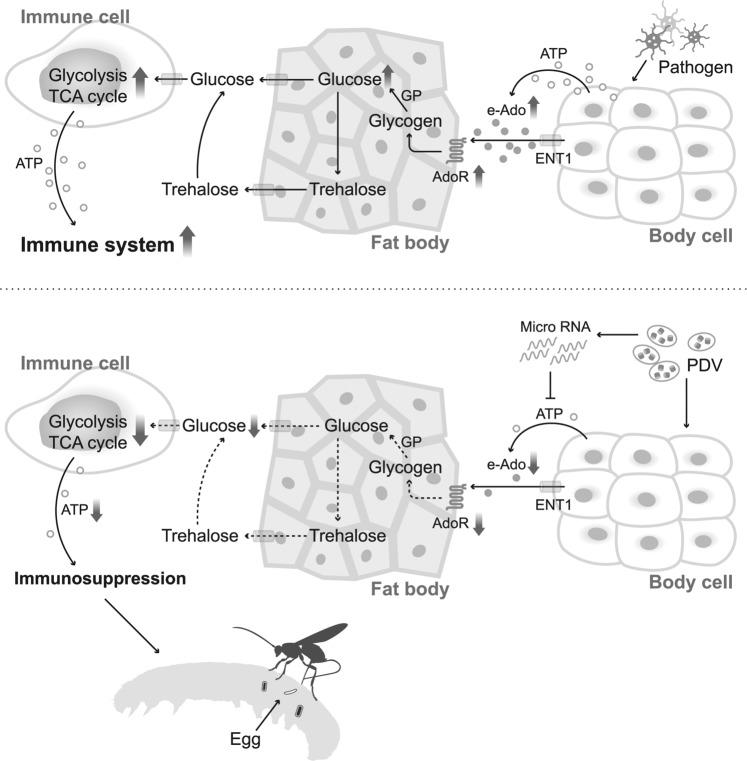
Schematic diagram of changes in carbohydrate metabolism after SmBV infection of *S. litura*. Top: Normal means in carbohydrate metabolism after pathogen infection in *Drosophila*. Pathogens cause the fat body to release large amounts of glucose to the hemocytes to promote carbohydrate metabolism. This provides energy to the immune system for pathogen defense. Bottom: Changes in carbohydrate metabolism after SmBV infection of *S. litura*. SmBV inhibits the adenosine synthesis pathway, causing decreased extracellular adenosine (e-Ado) concentration. This affects carbohydrate metabolism in hemocytes, ultimately leading to immunosuppression and enabling wasp eggs to successfully grow in *S. litura*.

Previous studies have demonstrated that PDV is crucial in immunosuppression upon wasp infection; the exact mechanisms are unclear. Our results demonstrate that SmBVs are able to suppress the host carbohydrate metabolism and immune responses by negatively regulating extracellular adenosine signaling (Fig. 8). To our knowledge, this is the first description of the mechanism by which viruses inhibit the host immune system by manipulating adenosine pathway which further compromises the host energy metabolisms.

## Methods

### Insects

*S. litura* were reared in cages and kept in an environmental chamber at 29 ± 1 °C with a light:dark cycle of 12:12 h. The formula of artificial diet was from a previous study^54^. *S. manilae* were reared in growth chambers at 26 ± 1 °C with a light:dark cycle of 12:12 h; 15% glucose was used for feeding^17^.

### Virus and cell

Purified SmBV was obtained by first resecting the calyxes of adult female *S. manilae*. The calyxes were then placed in TC-100 medium at 4 °C and homogenized using a pestle and mortar. The homogenate was centrifuged at 3000 rpm for 5 s. Then, the samples were removed, the supernatant was aspirated and a 0.45-μm syringe filter was used for purification. Finally, qPCR was used to quantitate virus copy numbers^55,56^. AcMNPV was cultured using the IPLB-Sf-21 cell line and qPCR was used to quantitate virus titers^57^. *S*. *litura* SL1A cells were cultured in TC-100 medium (USBio) containing 10% FBS (Gibco BRL) and cultured in a 26 °C incubator. SL1A cells (2 × 10^5^) were preincubated for 2 hours with Dipy (20 μM), 2DG (10 mM), or adenosine (100 μM). Subsequently, the cells were infected with AcMNPV at a multiplicity of infection (MOI) of 1. The supernatants were harvested to detect viral titers at 48 hours post-infection.

### Phagocytosis assay

Fourth-instar larvae were injected with SmBV (1 × 10^5^ copies/larva). After 36 h, hemocytes (4 × 10^4^ cells/well) were collected and FITC-labeled *E. coli* (4 × 10^4^ cells/well) with TC-100 culture medium were added in a total volume of 100 μl. The cells were cultured at room temperature for 60 min and subsequently washed 2–3 times with 10% phosphate-buffered saline (PBS). Then, 50 μl of 0.4% trypan blue was added and the cells stained for 10 min before washing 2–3 times with 10% PBS. Next, 4% formaldehyde was added and incubated for 30 s to fix the samples. Finally, 50 μl of DAPI (Thermo Fisher) was added, and the cells were incubated for 30 min and washed 2–3 times with 10% PBS. The proportion of FITC-labeled *E. coli* and hemocytes was counted by fluorescence microscopy^58^.

### Nucleic acid extraction and RT-qPCR

*S. litura* tissues were extracted using Trizol (Geneaid); detailed procedures have been described previously^59^. Briefly, a PrimeScript^TM^ RT Reagent Kit (Takara) was used for cDNA synthesis; 500 ng of RNA was collected and 2 μl of 5 × PrimerScript^TM^ buffer, 0.5 μl of PrimerScript RT Enzyme Mix I, 0.5 μl of Oligo dT primer, and 0.5 μl of random hexamers were added. A spectrophotometer (Nanodrop 2000) was used to measure the optical density of the RNA (260/280 nm). SYBR green (Bioline) and an ABI Plus One real-time system (StepOnePlus, Applied Biosystems) were used for qPCR. All the gene expression levels were normalized to the expression level of *18S*. A list of primer sequences used in this study is given in Table S1.

### Glycogen, glucose, and adenosine measurement

The levels of glycogen, glucose, and adenosine were determined in *S. litura* hemolymph using colorimetric methods with a glycogen assay kit (MET-5022, Cell Biolabs Inc.), glucose assay kit (Cell Biolabs Inc.), and adenosine assay kit (Cell Biolabs Inc.), respectively. Detailed procedures have been described previously^60^.

### Encapsulation assay

Hemolymph samples from fourth-instar larvae were collected and each mixed with 1 ml of Pringle’s saline^61^. The hemocytes were spun down by centrifugation at 500 × *g* at 4 °C for 5 min. After washing the hemocytes two times with Pringle’s saline, they were resuspended in 1 ml of saline; a hemocytometer was used to calculate the hemocyte density. Fifty microliters of the suspended hemocytes and 5 μl of Sephadex A-25 beads were added to each well of a 96-well plate^62^. Parafilm was used to seal the plates before incubation at 26 °C for observation of the encapsulation status.

### ATP analysis

The ATP level was quantitated by an ATP Determination Kit (A22066, Invitrogen). Ten microliters of hemolymph and 90 μl of reaction solution containing ddH_2_O, reaction buffer, dithiothreitol (DTT), D-Luciferin, and luciferase were mixed in a total volume of 100 μl. After reaction in the dark for 15 min, a luminometer was used to measure the luminescence at an absorbance wavelength of 560 nm. A standard curve was used to convert luminescence into ATP content.

### Adenosine feeding test

Newly hatched *S. litura* were given an artificial diet containing 1.65 mM or 5mM adenosine. After 7 to 8 days, eighty second-instar larvae were infected by ten *S. manilae* females for 48 h. The number of *S. manilae* that successfully pupated was calculated and regarded as viable *S. manilae* under host immune responses.

### Statistical analysis

The CT values obtained from the qPCR were used to obtain relative expression levels using the 2^−ΔΔ*Ct*^ formula; *18S* was used as the reference gene^56,63^. Each treatment group was compared with the control; Student’s t-test was used to test for significant differences. A heat map was plotted using the logarithm of the standardized *Ct* values (Log_10_) using Microsoft Excel^59^.

## Supplementary information

Dataset 1.
