# Supplementary material for: Snellenius manilae bracovirus suppresses the host immune system by regulating extracellular adenosine levels in Spodoptera litura
Source: Sci Rep. 2020 Feb 7;10:2096. doi: 10.1038/s41598-020-58375-y (PMC7005799; doi:10.1038/s41598-020-58375-y)
Supplement: Supplementary file 1 — Dataset 1. [file 41598_2020_58375_MOESM1_ESM.doc]

***Snellenius manilae* bracovirus suppresses the host immune system by regulating extracellular adenosine levels in *Spodoptera*** ***litura***

Yuan Chang^†^, Cheng-Kang Tang ^†^, Yu-Hsien Lin, Chih-Hsuan Tsai, Yun-Heng Lu and Yueh-Lung Wu^*^

**Supplementary data**

**Table S1. The list of qPCR primers**

| **Primer Name** | **Primers (5’→3’)** |
| --- | --- |
| 18S | F: 5’-GTAACCCGTTGAACCCCATT-3’  R: 5’-CCATCCAATCGGTAGTAGCG-3’ |
| Toll | F: 5’-CAATGGAATGGCTGCAGACA-3’  R: 5’-CCGATCTGGTTCGTGAATGG-3’ |
| Cecropin | F: 5’-TCGGCGTGCTTGGACAA-3’  R: 5’-CGTTGGACAAATGAGGATGATG-3’ |
| AdoR | F: 5’-GTGTTTCGGTGGATCGTTACTG-3’  R: 5’-ACCCAGCAGACGGAGATAATACC-3’ |
| Adgf | F: 5’-TTCGCTTCCTTCGTCGTCTG-3’  R: 5’-ATAGCGCGGGTGATCGAACG-3’ |
| GP | F: 5’-CATCGCCGACGTATTGCTT-3’  R: 5’-TGGTTCTGGTAAACACTGGAGACTT-3’ |
| Tre | F: 5’-GTGGTGTGTTTTTGTTGCGATT-3’  R: 5’-CAGCGGGCCATGACAGTAG-3’ |
| Pgi | F: 5’-TGCCTTCTACCAACTCATACATC-3’  R: 5’-CGTCAGCAGTCTTCCCCTTC-3’ |
| Pfk | F: 5’-GGCAGATCAAGTATTCATTCCC-3’  R: 5’-TCAGCACCCATACGACAACC-3’ |
| Tpi | F: 5’-ACCCCACAGCAGGCTCAAG-3’  R: 5’-CACCATACTGAATACGCACAGAGTT-3’ |
| Gadph | F: 5’-CGCAGCTAACTCGACATCAGG-3’  R: 5’TGTTTCAGTCCAAGCACAACG-3’ |
| Pglym | F: 5’-AGTGATAACCGCAGCCAACC-3’  R: 5’TGAAGAAGGAGCCCCGATAC-3’ |
| Eno | F: 5’-CAGCAGAAGGAGATTGACGA-3’  R: 5’GCCAAGTGTTTGTATAGGGGTA-3’ |
| Ldh | F: 5’-AGCATGGATCCGCCTTTTTA-3’  R: 5’CTCACGCCGGCTGCTATC-3’ |
| Cs | F: 5’-TCAGCCAACATTCCATCCAC-3’  R: 5’TCGGCACTCAAACCTCGTAG-3’ |
| Idh | F: 5’-CGTCCGTTGATGAGGCTGTT-3’  R: 5’CCACTTCCACGCTGACTTGA-3’ |
| Scs | F: 5’-GACACTGTGCGTCGGTATTGG-3’  R: 5’GTCCAGGGCACCTCCTTTTC-3’ |
| Ecto-NTPDase | F: 5’-TAGTCCACGGCAGTTCCTTC-3’  R: 5’-ATCGAGAATCCGTCTCCGCC-3’ |
| Ecto-5’-nucleotidase | F: 5’-AACGCTAGCGGTGATGTCGC-3’  R: 5’-TACCAGGTAAGCGACCCTCG-3’ |
| ENT1 | F: 5’-GGTAACAACCTGCCAAATGACA-3’  R: 5’-CTTCGCCGTGATGAACATGT-3’ |
| SmBV-C | F: 5’-AGCGGTATTTTGTTCCATTGTAGTT-3’  R: 5’-TGCCGCACCTTACAAGGATA-3’ |

**Fig. S1. The list of the targeting of SmBV miRNAs to the adenosine synthesis and metabolic pathways**.

**Supplementary Materials and Methods**

**miRNA target prediction**

To identify miRNAs that target **adenosine synthesis and metabolic pathways**, an open-source software based on the miRanda algorithm was employed to predict miRNA targets (John et al., 2004).

**References**

Chen, Y.W., Wu, C.P., Wu, T.C., Wu, Y.L., 2018. Analyses of the transcriptome of Bombyx mori cells infected with either BmNPV or AcMNPV. Journal of Asia-Pacific Entomology 21, 37-45.

John, B., Enright, A.J., Aravin, A., Tuschl, T., Sander, C., Marks, D.S., 2004. Human MicroRNA targets. PLoS Biol 2, e363.
